# Disparities in Heart Failure Epidemiology, Diagnosis, Management, and Outcomes

**DOI:** 10.7759/cureus.91788

**Published:** 2025-09-07

**Authors:** Amy R Yan, Pratyaksh K Srivastava, Darko Vucicevic, Manyoo A Agarwal, Boback Ziaeian, Gregg C Fonarow, Ali Nsair, Negeen Shahandeh

**Affiliations:** 1 Cardiology, The Bishop’s School, La Jolla, USA; 2 Cardiology, Ahmanson-UCLA Cardiomyopathy Center, University of California Los Angeles, Los Angeles, USA; 3 Cardiology, Cardio-Oncology Program, Cleveland Clinic Abu Dhabi, Abu Dhabi, ARE

**Keywords:** healthcare disparities, heart failure hospitalization, heart failure outcomes, management of heart failure, racial disparity, sex disparity

## Abstract

Heart failure (HF) disproportionately impacts minority groups in the United States. Among minorities living with HF, disparities exist in treatment, access to advanced therapies such as durable left ventricular assist devices and heart transplantation, and outcomes. Reasons for observed disparities range from the prevalence of traditional cardiovascular risk factors to social determinants of health, including housing, access to healthcare, and socioeconomic status. Clinical practice guidelines frequently are unable to address these observed disparities, and clinical trials often underrepresent minority populations. Custom programs tailored toward addressing disparities in HF care are needed to help improve outcomes in minority populations.

## Introduction and background

Heart failure (HF) is defined as a condition in which patients develop HF signs and/or symptoms that are due to a cardiovascular functional or structural abnormality in conjunction with either high levels of natriuretic peptides (hormones that regulate fluid balance) or pulmonary/systemic congestion of cardiac origin [1]. HF is further classified based on the left ventricular ejection fraction (LVEF), with LVEF of ≤40% defined as HF with reduced ejection fraction (HFrEF), LVEF of 41-49% defined as HF with mildly reduced ejection fraction, and LVEF of ≥50% defined as HF with preserved ejection fraction (HFpEF) [1]. HF is common, with a high prevalence in the United States (US) [2]. Similar to other cardiovascular diseases (CVDs), HF disproportionately affects different racial/ethnic groups [2]. Here, we evaluate the impact of race/ethnicity on various aspects of HF, including epidemiology, diagnosis, management, and outcomes.

## Review

HF epidemiology

HF is common in the US, with an estimated 6.7 million Americans carrying a HF diagnosis. Projections estimate that this number will rise to 8.5 million by the year 2030 [2]. Various large population health cohorts have helped elucidate the current burden of HF in the US [3-8].

Lifetime Risk of HF

The overall lifetime risk for developing HF is high and varies according to race and cohort studied. The Framingham Heart Study was established in 1948 to study the causes of heart disease and stroke. Since its creation, it has followed its enrollees every two years to study the impact of different risk factors on the development of CVD and stroke [3]. In the first epoch of the cohort, which reported on data from 1965 to 1989, the lifetime risk of developing HF was estimated at 19%. In the second epoch (1990-2014), the risk was estimated at 23.7% [3]. In this latter epoch, the risk of developing HFpEF was 19.3% and 14% for HFrEF [3]. In a large analysis of 39,578 participants from three large population health cohorts (the Chicago Heart Association Detection Project in Industry (CHA), the Cardiovascular Health Study (CHS), and the Atherosclerosis Risk in Communities (ARIC)), the risk of developing HF was found to differ by race. In CHA, the lifetime risk of HF was 30.2% in White men, 20.1% in Black men, 32.3% in White women, and 23.7% in Black women [4]. In ARIC, the risk was 19.1% in White men, 21.3% in Black men, 13.4% in White women, and 23.9% in Black women [4]. Pandey et al. analyzed the lifetime risk of HF in 12,417 enrollees > 45 years old from the CHS and Multiethnic Study of Atherosclerosis (MESA) cohorts. In this group, the risk of developing HF was 25.9% in non-Black patients vs. 22.4% in Black patients [5]. The overall risk of HF was higher in men (27.4%) than in women (23.8%) [5].

Prevalence of HF

Data from the 2017-2020 National Health and Nutrition Examination Survey (NHANES) estimate that 6.7 million Americans (age >20 years) currently carry a diagnosis of HF, with increasing burden tied to older age (fourfold greater prevalence in those ≥65 vs. <65) [6]. In the NHANES data from 1999 to 2018, the prevalence of HF increased from 19/1,000 participants in 1999 to 26/1,000 participants in 2017, though this change was not felt to be significant [6]. Among Medicare beneficiaries, a significant increase from 162/1,000 (2004) to 172/1,000 (2013) was observed [7].

**Figure 1:**
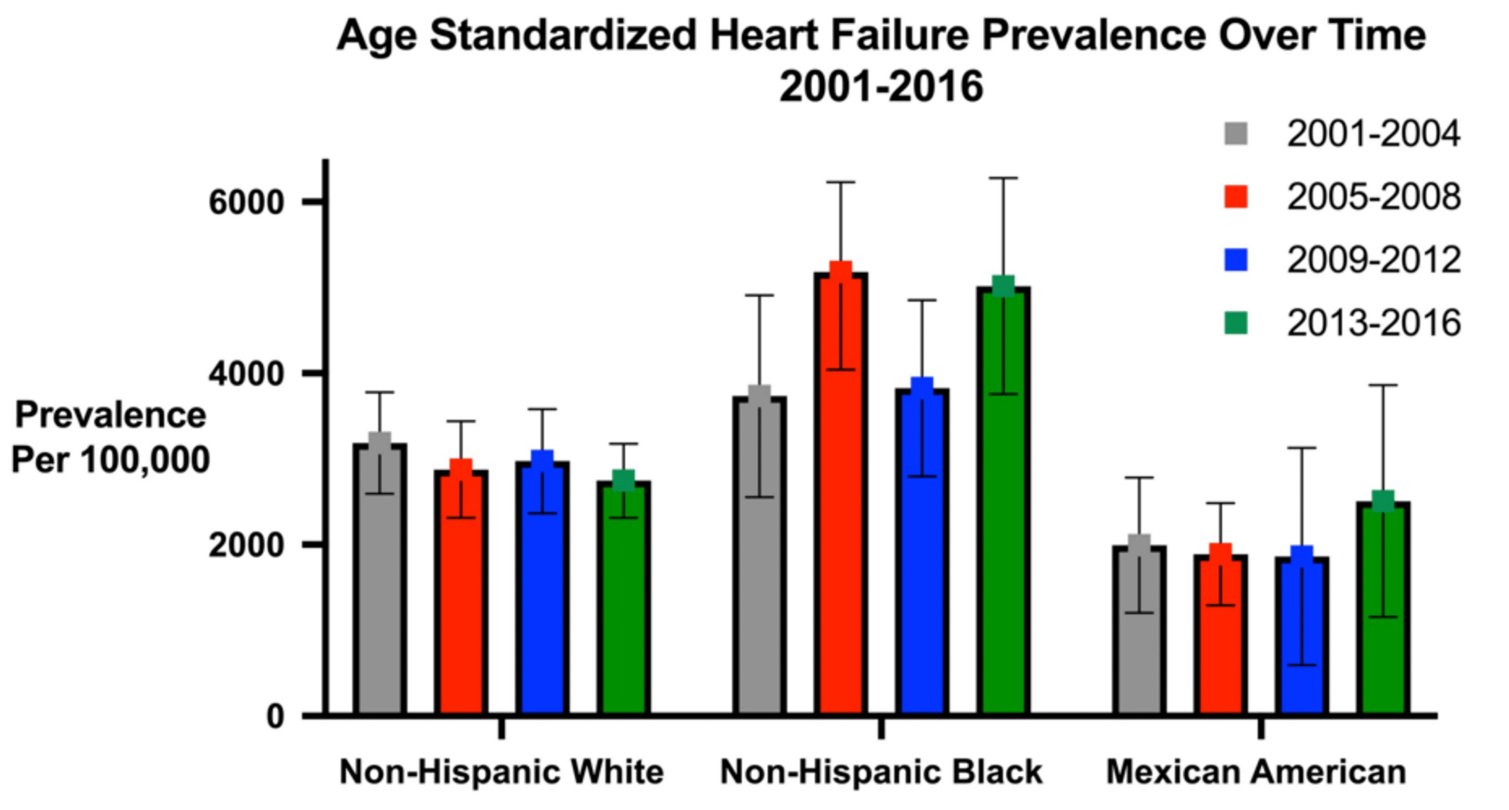
Age-standardized heart failure prevalence over time (2001-2016) Age-standardized heart failure prevalence over time (2001-2016) from the National Health and Nutrition Examination Survey. Graph made using the data from Rethy et al. [8].

In NHANES 2001-2016 data, the prevalence of ambulatory HF among non-Hispanic Black individuals increased over time from 3,733 per 100,000 people between 2001-2004 to 5017 per 100,000 people between 2013-2016 [8]. Of note, the prevalence was overall unchanged in the other racial groups, including among Mexican Americans and non-Hispanic White adults [8]. When stratifying the cohort by age, the prevalence of HF was similar across all racial groups for those >65 years. In those aged 35-64 years between 2013-2016, however, non-Hispanic Black adults had a higher prevalence of HF than non-Hispanic White adults (3864/100,000 vs. 1297/100,000 between 2013-2016) (Figure 1) [8].

As elucidated above, some studies appear to demonstrate a higher lifetime risk of HF in White patients, while other studies show that the prevalence of HF is higher in Black patients. This paradoxical finding may be explained by different study cohort time periods and makeup. The majority of the lifetime risk studies were performed in cohorts that began enrollment many decades ago and were followed through the early 2000s (CHA enrolled between 1967-1973 and followed until 2003; ARIC enrolled between 1987-1989 and followed until 2005; CHS enrolled between 1989-1993 and followed until 2004) [4]. These lifetime cohorts also had a relatively low percentage of minority patients (Black patients made up 10% of CHA, 27% of ARIC, and 16% of CHS cohorts) [4]. The incidence studies, on the other hand, were done in more modern cohorts that are likely more representative of the current American population [6-8].

HF management

In a study of 126,670 patients conducted within the Veterans Affairs healthcare system, Witting et al. [9] found that racial and ethnic minority patients newly diagnosed with HFrEF had similar rates of guideline-directed medical therapy (GDMT) prescription compared to White patients. In fact, treatment initiation rates were similar or higher among minority groups [9]. However, social and geographic disparities affected access to optimal care. Patients residing in socially vulnerable areas experienced a 3.4% reduction in angiotensin-receptor neprilysin inhibitor (ARNI) use, while those living farther from specialty care were less likely than closer patients to receive at least 50% of target doses for beta blockers (BB, 4.0% lower) or renin-angiotensin aldosterone inhibitors (RAASi, 5.0% lower; all p<0.05), despite similar initiation rates [9].

In the ARIC surveillance study of 16,455 HFrEF patients followed from 2005 to 2014, only about 10% of hospitalized patients with HFrEF received optimal GDMT. In this particular analysis, optimal GDMT was defined as a combination of BB, mineralocorticoid receptor antagonists (MRA), and either an angiotensin-converting enzyme inhibitor (ACEI) or angiotensin receptor blocker (ARB) [10]. In this study, a higher proportion of Black patients received optimal therapy compared to White patients (11.1% vs. 8.6%, p<0.001) [10]. BB use exceeded 80% in both groups, but Black patients were more likely to receive ACEI/ARB (62.0% vs. 54.6%) and MRA (18.0% vs. 13.8%) [10]. Additionally, hydralazine and nitrate therapy were prescribed more frequently to Black individuals (21.8%) than to White individuals (10.1%) [10]. In a large cohort analysis of 49,399 HFrEF patients from the Get With the Guidelines Registry hospitalized with HF between 7/1/2021 and 6/30/2022, sodium-glucose cotransporter-2 inhibitor (SGLT2i) prescription at discharge varied by race. Specifically, Asian (17%) and White (18.4%) patients were prescribed SGLT2i less frequently at discharge than Black (23.3%) and Hispanic (20.1%) patients (p<0.01) [11].

Similarly, a recent analysis of 82,637 patients from the American Heart Association’s Get With The Guidelines Heart Failure registry used a Quadruple Therapy Optimization (QTO) score to assess the four foundational therapies for HFrEF. Two points were assigned each for BB, ARNI, MRA, and SGLT2i use at discharge, and one point was assigned for ACEI/ARB use at discharge. The maximum possible maximum score was nine. Results showed that Black patients had significantly higher QTO scores than non-Hispanic White patients, with an adjusted mean difference of 2.56 percentage points [12]. Hispanic individuals also had higher QTO scores, although to a lesser extent (0.71 percentage points, both p values <0.05) [12].

HF hospitalization

An analysis by Agarwal et al. [13] using data from the Nationwide Readmissions Database examined over 6.3 million HF-related hospital encounters between 2010 and 2017. The study revealed that rates of primary HF hospitalizations per 1,000 US adults declined from 4.4 in 2010 to 4.1 in 2013 and then increased to 4.9 by 2017 [13]. Similarly, post discharge HF readmission rates declined from 1.0 in 2010 to 0.9 in 2014 before rising to 1.1 in 2017 [13]. All-cause 30-day readmissions followed the same pattern, decreasing from 0.8 in 2010 to 0.7 in 2014, and then increasing to 0.9 in 2017 [13]. These trends were consistent across sex-specific subgroups. These findings suggest that, after an initial decline, HF-related hospitalizations and readmissions have risen again in the present day.

Racial and ethnic disparities remain notable in HF hospitalization rates. A retrospective cohort study by Savitz et al. [14] within the Kaiser Permanente Northern California system analyzed HF outcomes among patients from 2012 to 2016. The findings demonstrated that Black patients experienced a 28% higher rate of HF hospitalization compared to White patients (adjusted hazard ratio (HR): 1.28, 95% confidence interval (CI): 1.18-1.38), even though they had a 22% lower rate of all-cause mortality (HR: 0.78, 95% CI: 0.72-0.85) [14]. In contrast, Asian and Pacific Islander patients had lower all-cause hospitalization (HR: 0.89, 95% CI: 0.85-0.93) and mortality (HR: 0.75, 95% CI: 0.69-0.80), and Hispanic patients also experienced reduced mortality (HR: 0.85, 95% CI: 0.80-0.91) relative to White individuals [14]. These disparities persisted after adjusting for socioeconomic status, comorbidities, and care quality measures and were most pronounced among Black patients with reduced ejection fraction [14]. These results suggest that measurable clinical and socioeconomic factors do not fully explain the observed racial disparities in hospitalization and mortality [14].

**Figure 2:**
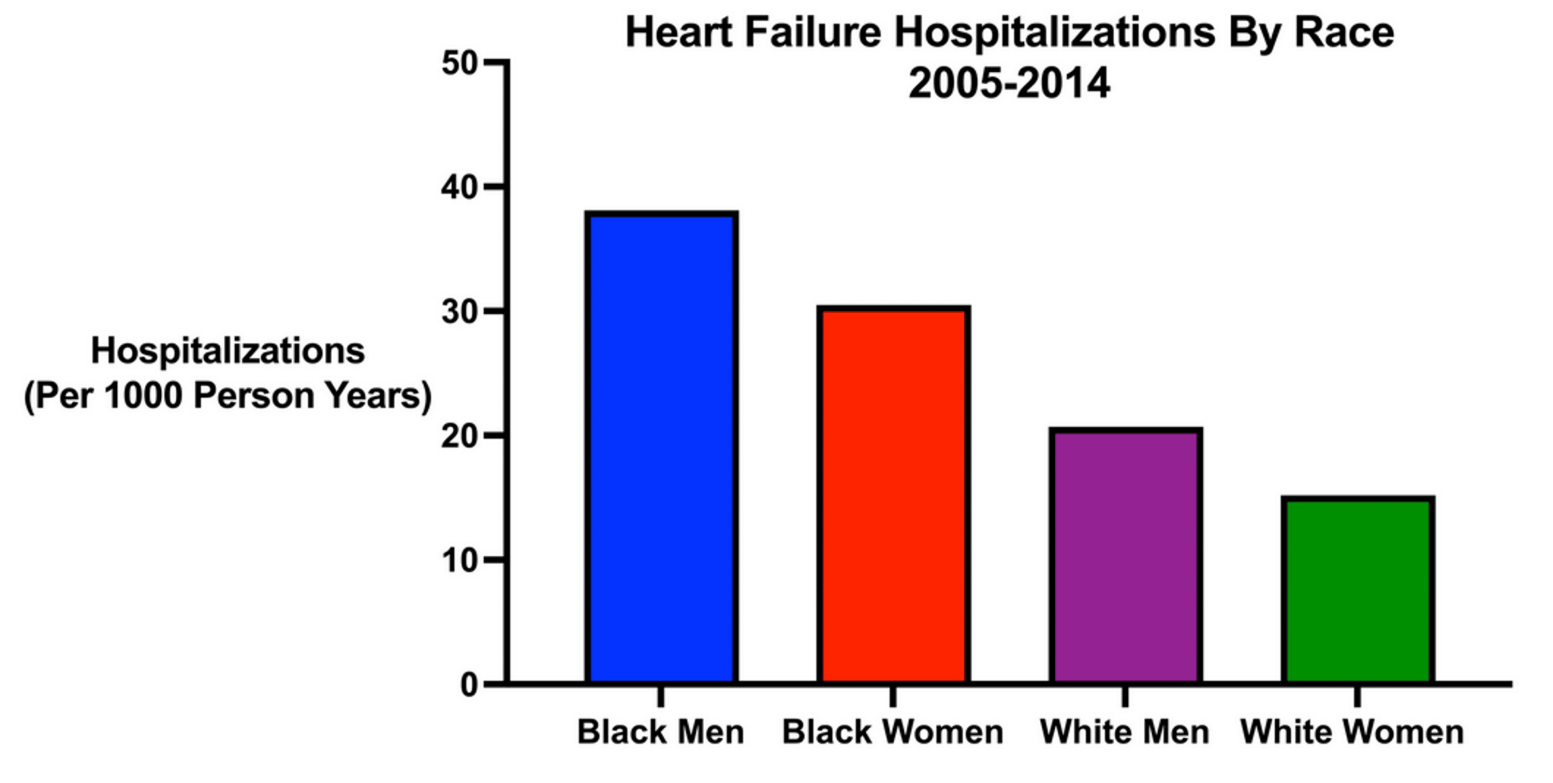
Heart failure hospitalizations by race (2005-2014) Heart failure hospitalizations by race (2005-2014) in the Atherosclerosis Risk in Communities (ARIC) Study. Graph made using the data from Chang et al. [15].

A 2014 ARIC study highlights similar racial disparities in acute decompensated HF (ADHF) hospitalizations. From 2005 to 2014, Chang et al. [15] analyzed over 40,000 weighted ADHF hospitalizations in adults aged 55 and older across four US communities. They found that HFrEF was more prevalent in Black and White men, whereas HFpEF was most common in White women [15]. ADHF hospitalization rates were the highest in Black individuals at 38.1 per 1,000 Black men and 30.5 per 1,000 Black women, rates substantially higher than White patients (Figure 2) [15]. These rates were found to increase over the study time period, with average annual percent change differing by race: Black men +3.7%, Black women +4.3%, White men +2.6%, and White women +1.9% [15]. Additionally, despite persistently high one-year mortality rates (~30%) across all demographic groups, only Black men and Black women saw significant mortality declines during the study period, with the most substantial improvements observed among Black women with HFpEF (-7.1% annual decline) [15].

HF device use 

**Figure 3:**
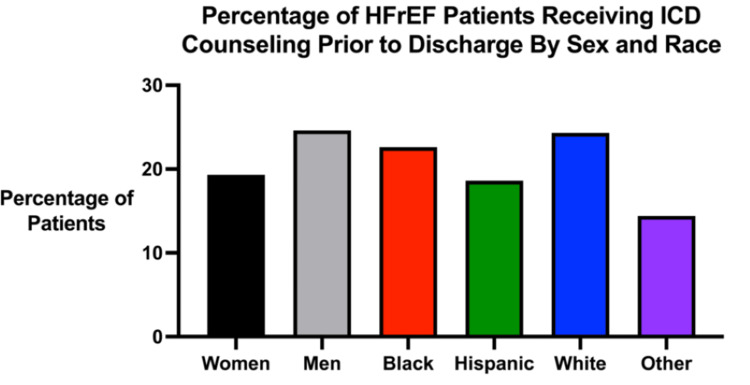
Percentage of HFrEF patients receiving ICD counseling prior to discharge by sex and race Percentage of heart failure with reduced ejection fraction (HFrEF) patients receiving implantable cardioverter defibrillator (ICD) counseling prior to discharge by sex and race. Graph made using the data from Hess et al. [16].

Despite guideline recommendations for the use of implantable cardioverter defibrillators (ICDs) in eligible patients with HFrEF, significant disparities in both counseling and implantation rates across minority groups exist. A study conducted by Hess et al. found that only 22.6% of eligible patients received predischarge counseling about ICDs, with women and racial/ethnic minorities receiving less counseling compared to White men [16]. Specifically, 19.3% of women were counseled compared to 24.6% of men (OR: 0.84, 95% CI: 0.78-0.91), and minority groups such as Black (22.6%), Hispanic (18.6%), and other non-White (14.4%) patients received less counseling compared to White patients (24.3%, all p<0.001) (Figure 3) [16]. While sex-based differences in receiving an ICD among counseled patients were not statistically significant, racial disparities persisted, with Black patients (OR: 0.70, 95% CI: 0.56-0.88) being less likely to receive an ICD compared to White patients [16].

Disparities in ICD use are also apparent among patients with hypertrophic cardiomyopathy (HCM), a condition with elevated sudden cardiac death risk that is managed with ICD therapy in select individuals [17]. Patlolla et al. [18] analyzed 23,535 adult HCM hospitalizations using the National Inpatient Sample from 2003 to 2014 and found that ICD implantation occurred in 16.8% of cases [18]. ICD use increased over time, from 11.6% in 2003 to 17.0% in 2014 [18]. Women had a 28% lower odds of receiving an ICD compared to men (OR: 0.72, 95% CI: 0.66-0.78), and patients of non-White race had a 13% lower odds compared to White patients (OR: 0.87, 95% CI: 0.79-0.96) [18]. Furthermore, women and non-White patients experienced statistically higher rates of device-related complications, longer hospital stays, and greater hospitalization costs [18].

Advanced therapy use

Advanced therapies, such as durable left ventricular assist devices (LVADs) and heart transplantation, offer life-prolonging technologies for patients with end-stage HF, yet racial and socioeconomic disparities impact equitable access and outcomes. In the Registry Evaluation of Vital Information for VADs in Ambulatory Life (REVIVAL) study, 377 ambulatory patients with chronic systolic HF, high-risk features, and no contraindication to LVAD were enrolled at 21 VAD centers over two years. Black patients were significantly less likely to receive LVAD or transplant compared to White patients (11% vs. 22%), despite having similar rates of preference for these interventions [19]. The hazard ratio for VAD/transplant among Black patients compared to White patients was 0.45, indicating a 55% lower likelihood of receiving these therapies even after accounting for factors such as HF severity, quality of life, social determinants of health, and patient preferences [19]. Notably, this underutilization did not correspond to statistically significantly higher mortality, although death rates remained high in this cohort (18% in Black patients and 13% in White patients) [19]. In a broader analysis of Medicare beneficiaries hospitalized for HF between 2008 and 2014, Cascino et al. [20] found that Black patients were 3.0% less likely than White patients to receive an LVAD, and women were 7.9% less likely than men to undergo LVAD implantation (p<0.05) [20]. Individual poverty and neighborhood deprivation were also associated with reduced use (2.9% and 6.7%, respectively), but these factors did not fully account for the observed racial disparities [20].

Disparities in access also appear present in the heart transplant evaluation and offer process. In a national analysis of transplant candidate data from 2018 to 2023, Breathett et al. [21] found that Black patients, in comparison to White patients, had lower odds of donor heart offer acceptance beginning with the very first offer (OR: 0.76, 95% CI: 0.69-0.84) [21]. This disparity persisted through the 16th offer, even after adjusting for candidate, donor, and other factors [21]. The cumulative incidence of acceptance was the highest among White women, followed by Black women, White men, and lowest among Black men (p<0.001) [21]. Notably, women were more likely than men to have their first offer accepted (OR: 1.53, 95% CI: 1.39-1.68), but this trend reversed for offers 10 through 31 [21].

Although listing rates for Black and Hispanic patients have improved over the years, similar disparities continue to persist. Chouairi et al. [22] analyzed over 32,000 transplant candidates from 2011 to 2020 and found that the percentage of Black and Hispanic patients listed for transplant increased over the decade, from 21.7% to 28.2% for Black patients and from 7.7% to 9.0% for Hispanic patients (both p<0.05) [22]. Despite this increase in listing, Black patients were significantly less likely to undergo transplantation than White patients (OR: 0.87, 95% CI: 0.84-0.90) and had a higher risk of post-transplant death (OR: 1.14, 95% CI: 1.04-1.24) [22]. Even after the 2018 allocation policy change, which increased transplant rates for all groups, Black patients continued to have lower transplantation rates compared to White patients (OR: 0.90, 95% CI: 0.79-0.99) [22].

HF outcomes

The National Centers for Disease Control and Prevention (CDC) Wide-Ranging Online Data for Epidemiologic Research (WONDER) shows that HF mortality declined from 1999 to 2012 for patients aged 35-84 years (78.7-53.7 per 100,000) but increased again from 2012 to 2017 (59.3 per 100,000) [23]. Even though mortality was higher in older adults (ages 65-84), the annual rate of increase since 2012 has been greater among younger adults (ages 35-64) [1,23]. Among individuals aged 15-44 years, the age-adjusted mortality rate rose from 2.36 per 100,000 in 1999 to 3.16 in 2019, with Black individuals and men experiencing the highest mortality rates in this age group [24]. In individuals 75 years and older, age-adjusted mortality declined from 141.0 per 10,000 in 1999 to 108.3 per 10,000 in 2012 and then increased again to 121.3 per 10,000 in 2019, with the highest rates seen in non-Hispanic White individuals, followed by non-Hispanic Black individuals, non-Hispanic American Indians or Alaska Natives, Latinos or Hispanics, and non-Hispanic Asian/Pacific Islanders [25]. Additionally, rural areas consistently demonstrated higher HF mortality for both younger and older populations [25,26].

Post-transplant outcomes also reveal related disparities. In a study of 39,075 heart transplant recipients (1987-2009), Black patients were found to have a 34% increased risk of mortality (HR: 1.35, 95% CI: 1.21-1.47) compared to White patients, as well as an increased five-year mortality (35.7% vs. 26.5%) [27]. Black patients have also been shown to experience more graft-related complications, including rejection, failure, and allograft vasculopathy [27,28]. Among younger recipients 18-30 years old, a study of 22,997 heart transplant recipients from the Scientific Registry of Transplant Recipients (2005-2017) showed that Black patients had higher mortality compared to non-Black patients (HR: 2.3, 95% CI: 1.60-3.31) [29]. The disparities persisted even after accounting for socioeconomic status and other risk factors such as income, insurance, or education [29].

Determinants of HF disparities

As elucidated above, minority populations experience significantly worse HF morbidity, mortality, and outcomes when compared to non-minority groups. There are several possible explanations for these findings. These range from an increased burden of traditional cardiovascular risk factors, such as hypertension, diabetes mellitus, and physical inactivity, to social determinants of health, including lower socioeconomic status and decreased access to healthcare [30].

Socioeconomic inequality influences HF outcomes. Black and Hispanic populations are disproportionately represented in lower socioeconomic strata, which can be associated with access to lower-quality healthcare and difficulty accessing medication and preventative services [30-32]. For example, lower income is independently associated with increased HF risk and mortality [30,33,34]. These groups are more likely to live in racially divided and under-resourced neighborhoods due to historic and ongoing practices such as redlining and predatory lending [31]. Such environments are also linked to higher rates of hypertension, diabetes, and obesity, all risk factors for HF, even after accounting for individual-level characteristics [31,34,35].

Structural racism further contributes to the issue, manifesting through systemic barriers such as unequal access to specialty care and implicit bias among providers [30,36,37]. Various studies have found that Black patients are less likely to be referred for advanced therapies such as transplantation and are more likely to receive care in emergency rather than outpatient settings [22,30,38]. Even when hospitalized, Black and Hispanic patients are less likely to be admitted to cardiology services or receive care from cardiologists, negatively affecting outcomes [30,39].

Additionally, policy-level factors continue to exacerbate these disparities. For example, the Hospital Readmissions Reduction Program, though designed to improve care quality by penalizing hospitals with high readmission rates, does a poor job accounting for socioeconomic factors [40,41]. As a result, the program frequently penalizes “safety-net” hospitals that serve greater proportions of minority populations and socioeconomically disadvantaged patients, making it even more difficult for these individuals to access proper care [40,41].

Finally, clinical practice guidelines often lack nuance in addressing these systemic disparities: most HF guidelines are not tailored to account for the social and structural challenges that may affect treatment adherence and outcomes in minority populations [30]. Additionally, these guidelines typically emphasize strategies that are rooted in clinical trial evidence, yet minority groups, especially Black, Hispanic, and Indigenous populations, have historically been underrepresented in these trials [30].

## Conclusions

Disparities in HF prevalence, management, access to advanced therapies, and outcomes continue to persist in the US. Such findings are likely at least in part due to a complex interplay of cardiovascular risk factors and social determinants of health. Policy and further research are needed to develop tailored strategies to help address disparities observed among minorities with HF.
